# Play is a play, is a play, is a play… or is it? Challenges in designing, implementing and evaluating play-based interventions

**DOI:** 10.3389/fpsyg.2023.1034633

**Published:** 2023-04-03

**Authors:** Elena Bodrova, Deborah Jane Leong, Elena Yudina

**Affiliations:** ^1^Tools of the Mind, Denver, CO, United States; ^2^Moscow School of Social and Economic Sciences, Moscow, Russia

**Keywords:** social-emotional learning, play, classroom intervention, early childhood, children

## Abstract

When a social-emotional learning (SEL) intervention is implemented in an early childhood classroom, it often involves play. Some interventions even list play as its main component. However, the advocates of play arguing for the return of play in early childhood education (ECE) classrooms still have difficulty convincing the proponents of more rigorous academic instruction. These proponents cite research pointing to the insufficient evidence of the positive effect of play on children’s short- and longer-term social, emotional, cognitive, and behavioral outcomes as well as their overall well-being. We believe that there are multiple issues with play-based interventions’ design, implementation, and evaluation that might account for this insufficient evidence. In our paper, we discuss the numerous ways play does (or does not) feature in SEL interventions and how it might affect the outcomes of these interventions. We also examine the methodological challenges of having child-controlled play as a component of an SEL intervention. While we are not proposing a specific protocol for re-evaluation of the results of existing interventions, we outline some ways such re-evaluation can be possible in the future, along with the development and evaluation of new play-based SEL interventions.

## 1. Introduction

With early childhood being the formative period for the development of children’s social and emotional skills, it is now recognized that the programs targeting these skills in the early years have the greatest potential to promote children’s well-being and healthy development (Blewitt et al., 2018). Over the past decades, the number of these programs has been growing, with the programs taking on various formats, from narrowly targeted social-emotional learning (SEL) interventions to the comprehensive early childhood education (ECE)^1^ curricula that embed SEL support in multiple materials and activities.

To assist educators in the adoption process, several review papers have been published that compare various SEL programs in their effectiveness in strengthening children’s social and emotional competencies and preventing challenging behaviors (Joseph and Strain, 2003; Dunlap and Powell, 2009; O'Conner et al., 2017; Blewitt et al., 2018; Yang et al., 2019; Murano et al., 2020). Authors identified several characteristics shared by successful programs, such as well-defined and systematically addressed social and emotional target skills, teacher professional development and ongoing support, and the continuity in supporting social and emotional skills between school, family, and community (O'Conner et al., 2017). All these characteristics do not seem to be unique to ECE and could be applied to the interventions implemented at any grade level.

These reviews leave us with a question: If we assume a qualitative difference between different periods in child development, would not this imply that there are some unique features of young children’s learning and development and that these unique features should be reflected in the design and implementation of the SEL interventions? We suggest that one of these unique features of early childhood is children’s engagement in play as a freely chosen and intrinsically motivated activity controlled by the players. While humans engage in various forms of play way beyond early childhood (Van Vleet and Feeney, 2015), it is the early years when the impact of play on development is the greatest. The acknowledgment of the critical importance of play for young children is based on evidence from diverse fields, from evolutionary psychology (Greve and Thomsen, 2016) to child development (Vygotsky, 1967; Hewes, 2014) to pediatrics (Ginsburg, Committee on Communications, and Committee on Psychological Aspects of Child and Family Health, 2007; Yogman et al., 2018) and is reflected in the UN Convention on the Rights of the Child (Hodgkin and Newell, 2007). It, therefore, seems logical to include play as a context for SEL development in early childhood.

The words ‘early childhood’ and ‘play’ have been almost synonymous for so long that very few scholars question this connection. The pioneering work of Mildred Parten (1932) made the connection between play and children’s social development universally recognized. This tradition of associating changes in children’s play with their social development, however, stands in contrast to the fact that play is rarely mentioned in the reviews of the research on SEL programs and is never discussed as one of the active ingredients of these programs. In addressing child development from the perspective of the ‘whole child,’ this omission is problematic. In this paper, we discuss commonly used definitions of play, explore the different ways play is used in SEL interventions, examine reasons why play might be omitted or underused as a specific SEL strategy, and address ways in which developers of the SEL interventions might look at play in relation to the different aspects of their interventions.

The topic of play in early childhood has recently re-surfaced in the context of the increasing academic pressure experienced by preschool and kindergarten teachers leading to the virtual disappearance of play from ECE classrooms and the entire culture of childhood (Gray, 2011; Belknap and Hazler, 2014; Wohlwend and Peppler, 2015; Barblett et al., 2016; Bassok et al., 2016; Whitebread, 2017; Wasmuth and Nitecki, 2020; Digennaro, 2021). Given that many of today’s children spend a significant portion of their waking hours in some kind of a school or center environment and that their opportunities to engage in play outside of this environment are diminishing (Singer et al., 2009), it is imperative to make sure that play becomes a critical part of any early childhood intervention and especially an SEL intervention.

## 2. Play: What’s in the word

One of the reasons that play is not prominently featured in interventions that attempt to promote children’s social and emotional development is the elusive nature of play: while everyone seems to have an intuitive understanding of how play is different from non-play, it is hard to convert this understanding into well-defined characteristics of play that can be reliably measured and manipulated. While the educational field has not come to a single definition of play, there seems to exist some agreement about the features of an activity that qualifies as play: it must be pleasurable, process-oriented, intrinsically motivated, meaningful, iterative, and controlled by a child (Canning, 2012; Zosh et al., 2017, 2022). The activity possessing these qualities is frequently described as *spontaneous*^2^ or *free* play (Hewes, 2014) to distinguish it from other activities that retain some degree of playfulness but are not entirely intrinsically motivated or child controlled.

Those other ‘playful’ activities are given such names as *guided play* or *purposeful play* to emphasize the fact that this kind of play is controlled (at least partially) not by a child but by an adult (Hirsh-Pasek and Golinkoff, 2008; Weisberg et al., 2016; Yu et al., 2018; Allee-Herndon and Roberts, 2021). The adult-initiated play category also includes ‘serious games’ (Zosh et al., 2018), sometimes called *structured play* (Healey and Healey, 2019). The division between free play and adult-involved play is not static: an adult may intervene in children’s play to infuse it with the academic content without completely taking it over. Zosh et al. (2018) attempted to capture the heterogeneity of children’s playful experiences by conceptualizing play as a spectrum, with these experiences differing in terms of adult or child initiation and direction of play and the presence of a learning goal.

Authors sometimes combine free play, guided play, and games in a more general category of *playful* or *play-based learning* (Danniels and Pyle, 2018; Zosh et al., 2022). This kind of learning is argued to be preferable for young children as compared to learning in more ‘schoolified’ settings (Zosh et al., 2022). At the same time, when examining the role of play in social-emotional learning, it seems essential to unpack the concept of ‘playful learning’ and to identify the exact characteristics of a specific playful experience, such as the degree of adult-directedness or child agency. In our paper, we will reference these and other characteristics of play when discussing the use of play in SEL interventions.

In addition to the activities explicitly labeled ‘playful,’ many activities for young children in the SEL interventions are designed to have some play elements. Examples include children’s role-playing that follows a script of a social situation (Wee et al., 2022) or using teddy bears to help children express their feelings (Koplow, 2008). Unfortunately, based only on the researchers’ accounts, it is hard to determine whether these activities were considered ‘playful’ by children. Even young children can detect when adults offer them a learning activity under the guise of play, and children perceive an activity as ‘play’ when there is “an element of choice and sharing of control” (Jensen et al., 2021, 493).

## 3. Locating play in the space of SEL interventions

Different kinds of play and other playful activities can be a component of an SEL intervention. To maximize the role of these activities, it is important to identify them correctly and examine their relationship with specific social and emotional skills. Combining disparate programs under an umbrella of ‘play-based’ makes it difficult to unpack these programs’ effects on social and emotional development. We identified four ways play and the activities described as ‘playful’ are (or are not) included in the SEL interventions: (1) play is not included in the design of the intervention, thus making it ‘invisible’ for the intervention developers and researchers; (2) play is used in the intervention as the primary vehicle to promote SEL; (3) the intervention focuses on improving the quality of play, and (4) SEL is one of the areas targeted by a comprehensive play-based curriculum. For each of these four categories, we see different challenges in the design, implementation, and evaluation of the intervention associated with the way children engage in play.

### 3.1. Challenges in making ‘invisible’ play visible

Some interventions consist of a series of lessons, each teaching a specific skill, such as recognizing and labeling one’s own and each other’s emotions or inhibiting impulsive reactions (e.g., Domitrovich et al., 2007; Webster-Stratton and Reid, 2008). Teachers usually deliver these lessons in a large group setting (e.g., during circle time), and children then practice newly learned skills throughout their daily activities (Blewitt et al., 2018). In typical ECE classrooms, this practice would most likely occur during center time or other free play periods. Although play is not explicitly listed as a component of practice activities and therefore stays ‘invisible’ to the developers of the intervention, it is likely that it still figures in some way in what children are doing. As play within these classroom activities remains ‘invisible,’ it is unclear which of the activity’s quantitative and qualitative characteristics allow children to practice newly learned social and emotional skills. The quantitative characteristics include but are not limited to the overall duration of activity as well as the duration of uninterrupted activity, the number of children in the same center at a given time, the number of children entering and exiting a center during a specific time period, etc.

The choice of qualitative characteristics to examine depends on the specific aspect of social-emotional learning. For example, qualitatively different activities such as joint block-building and social pretend play provide different opportunities for developing communication, cooperation, and perspective-taking skills. If play is part of this context where young children’s social-emotional learning happens, it is crucial to make play ‘visible’ and take into account the elements that would make it more effective in supporting social-emotional skill development, such as the degree of children’s control over the flow of play, the existence of rules and the opportunities to establish new rules, etc. (Burdette and Whitaker, 2005; Hewes, 2014; Jarvis et al., 2014; Nicolopoulou and Smith, 2022).

While the literature on these interventions does not provide a description of play in the treatment classrooms, it often mentions the curriculum used (e.g., HighScope or Creative Curriculum) or the kind of setting (Head Start classroom, public school pre-kindergarten, etc.). A closer look at the classrooms may show us significant variations in the implementation of the same curriculum or in following the same program guidelines. For example, most preschool classrooms have a substantial portion of time on their schedule described as ‘free choice time’ or ‘center time.’ This time block is usually when most of the indoor play takes place. Whether or not children have ample opportunity to practice social and emotional skills in play depends to some degree on how this free choice time is managed.

There are at least two variables that, in our opinion, should be considered in planning an intervention regarding play that happens in the activity centers during the free choice time that serves as a context for SEL. The first variable is the time children spend in one center or one activity. In some early childhood programs, children spend the entire time engaged in play as several centers get integrated into a general play theme. In the others, children rotate from one center to the next, which leaves them with less than 20 min to spend in each center (Paulick, 2019), although it has been known for a long time that children need at least 30 min to engage in high-level play (Christie and Wardle, 1992). In their paper, Christie and Wardle make a compelling case for allocating more time to uninterrupted play as they demonstrate the complexity of children’s behaviors in the preparatory stages of high-level sociodramatic or constructive play. These behaviors are necessary for play to reach this level. The authors also address the issue of children needing access to all activity centers by suggesting closing centers on a rotating basis for children to have “several long play periods per week rather than short daily ones” (p. 30).

Many kindergarten classrooms have activity centers that do not necessarily allow for sociodramatic or constructive play. Instead, centers are where children practice academic skills (Bassok et al., 2016). The kind of play most common in kindergarten is board games, but sometimes the access to the games is contingent on children completing the academic assignments, so the actual time spent playing may vary.

Another variable to consider is the degree of teacher-directedness of the activities available in the centers during ‘play time.’ Contrary to its name, in many programs, the ‘free choice time’ provides children with limited choices as some centers get converted to small group teacher-directed activities (Paulick, 2019). While teacher-directed activities are associated with children’s gains in academics (De Haan et al., 2014; Goble and Pianta, 2017), it is the child-managed activities that contribute to the development of such social-emotional skills as inhibitory control, attention, and resilience to stress (Burdette and Whitaker, 2005; Hewes, 2014; Goble and Pianta, 2017).

Making play (or the absence of play) in ECE classrooms ‘visible’ would help the developers of SEL interventions in the planning stages as they decide if their intervention has a good chance of producing its desired outcomes when implemented in these classrooms. It might also provide an additional lens through which they can evaluate the implementation results.

### 3.2. Challenges in using play as an instrument to promote SEL

The interventions that use play to promote social and emotional competencies vary in the type of play used and the target skills. The typical features of these interventions are their relatively short time span (usually several weeks), short duration of play activities (20–30 min), and a relatively high degree of adult-directedness of the play activities offered to children. Examples of these activities include movement games with increasingly more complex rules (McClelland et al., 2019), listening to and acting out the stories (Joseph and Strain, 2003; Mondi et al., 2021), and playing group games (Barrow et al., 2015). The outcomes of these interventions include emotion control, self-regulatory skills, and a decrease in behavior problems (Healey and Healey, 2019). Children’s social and emotional skills progress is typically measured immediately after the intervention ends and sometimes several months later on various measures, including teacher reports and standardized tests. Most authors report that assessing the transfer of skills targeted by their intervention to children’s free play and other contexts falls outside the scope of their current research while at the same time acknowledging the importance of studying this transfer in the future.

The issue of the transfer from mostly adult-directed play used in these interventions to other classroom activities is especially important for such skills as self-regulation and emotion control. Are children able to apply newly learned skills in an activity that is completely child-initiated and child-controlled? Is there a gradual transition from adult regulation to self-regulation, and what are the activities that work best in facilitating this transition? With self-regulation and emotion control being a target of an SEL intervention, these questions need to be answered not only to determine the practical benefits of this intervention but also to inform the development of its new versions.

### 3.3. Challenges in improving the quality of play as a means to promote SEL

Over the past decades, evidence has been accumulating, indicating that children’s pretend play is experiencing a decline not just in its quantity but in its quality as well (Jarvis et al., 2014; Lewis, 2017; Smirnova and Gudareva, 2017). Today’s four-and five-year-olds are playing in a way more typical for younger children: their pretend scenarios are stereotypical, the use of props is non-imaginative, and they cannot sustain play for prolonged periods (Lemay et al., 2022). Researchers refer to this kind of play as ‘low-level’ or ‘immature,’ contrasting it with ‘high-level’ or ‘mature’ play that involves “elaborate group dramatizations and complex construction projects” (Christie and Wardle, 1992, p. 28). Immature play is associated with lower levels of various social and emotional skills, including self-regulation and cooperative behaviors (Slot et al., 2017). It is possible that this decline in the quality of play is one of the reasons that play-based interventions do not always produce expected results and that some scholars have started voicing their doubts about the validity of assigning play the central place in promoting health, learning, and development of young children (Lillard et al., 2013).

This observable proliferation of less developed or immature play prompted the researchers to design interventions to promote the quality of child-controlled play. The interventions promoting mature play were able to elevate levels of play and improve children’s emotion control, executive functions, and self-regulation (Diamond et al., 2007; Blair and Raver, 2014; Perren et al., 2019; Richard et al., 2021; Adam et al., 2022). This group of interventions faces additional challenges associated with the complex nature of child-controlled play.

The need to maintain a delicate balance between teacher support and children’s independence presents a significant challenge for assessing play, as many of the skills contributing to mature play, such as background knowledge or problem-solving, seem to belong to the unconstrained category: they cannot be taught directly but instead develop gradually through varied experiences (McCormick et al., 2021) Assessing unconstrained skills presents a challenge not only for the play researchers but for the entire field of child development and learning (Dowd and Thomsen, 2021). In addition, using some of the existing play measures does not provide an accurate picture of the status of play: often, what is being observed and measured is not yet actual child-controlled play, but most likely, it is still adult-guided play involving substantial teacher support. Using more fine-grain measures, such as teacher-child interactions in play, may establish the relationships between these interactions and children’s social-emotional learning, such as their use of regulation-related skills (Moreno et al., 2017). Yet another approach is to assume that in an ECE classroom, children’s play always reflects both: the adult support and the children’s ability to benefit from this support. This approach yielded measures of play that combine children-level variables with teacher-level variables (Leong and Bodrova, 2012; Germeroth et al., 2019). These instruments provide an overall measure of play maturity in the context of scaffolded teacher-child interactions.

Another challenge lies in the timing of the evaluation. It takes a long time for children’s play skills to fully develop and solidify, especially if these children initially have immature play skills. As a result, evaluations scheduled too early can yield lower-than-expected outcomes. Providing play support for an entire year or even longer, as well as monitoring levels of play maturity, will hopefully allow researchers and curriculum developers to collect necessary evidence linking the development of mature play to the growth of children’s social and emotional skills.

### 3.4. Challenges in using a play-based curriculum to promote SEL

The variations in the use of the term *play* result in the variability of what counts as a play-based curriculum. Many such curricula self-define as play-based by contrast, i.e., implying that they are not using didactic modes of instruction but instead engage children in play and games without specifying what these play and games are (Reynolds et al., 2011). Some curricula implement play-based methods in teaching specific subject matters (mostly literacy and math), while others generalize the play-based approach to all areas of child development. There are several theoretical models underlying play-based curricula, including the idea of combining playful activities with varying degrees of adult-directedness (Zosh et al., 2022), infusing academic objectives in promoting children’s pretend play (Fleer et al., 2017), and co-constructing play as Developmental Education (Van Oers and Duijkers, 2013).

At the same time, many commercially available and teacher-designed curricula self-identify as ‘play-based’ by merely describing the classroom setup (activity centers and not desks). As we discussed in the ‘invisible play’ section, the presence of centers does not necessarily translate into the quantity or quality of play in these centers. Knowing not only the *intended* curriculum but also the *enacted* one (Porter et al., 2001) might help determine the optimal fit between the SEL intervention and the context where it gets implemented.

Regarding the evaluation, a play-based curriculum faces all challenges discussed in the previous sections. In addition, these curricula are currently expected to deliver results not only in the general areas of child development but also in foundational academic competencies. With the academic (mostly ‘constrained’) skills more amenable to change short-term (Casbergue, 2010; McCormick et al., 2021), play-based curricula often find themselves at a disadvantage compared to skill-based curricula when children get assessed on discrete academic skills only. Unfortunately, it is becoming harder and harder to use previous studies, including the classic Perry Preschool study (Schweinhart, 2019), in defense of play-based programs because of the changes in school readiness expectations. Conducting new longitudinal studies with children repeatedly assessed on both cognitive and noncognitive (Heckman, 2011) skills may help determine not only the immediate but also long-term effects of play-based instruction during early childhood. These effects may be latent, manifesting many years later, or they may interact with other educational or parenting factors resulting in a cumulative impact on child development (Maggi et al., 2010).

Additional evaluation challenges are associated with quality rating systems and other classroom environmental rating scales used to evaluate early childhood programs. Although these instruments position themselves as ‘curriculum independent,’ they often value classroom practices that promote only one specific type of play at the expense of other playful activities. For example, a widely used Early Childhood Environmental Rating Scale (Harms et al., 2014) distinguishes between a ‘play area’ defined as a space with pretend play materials, and ‘interest centers’ such as blocks or art. Such distinction implies that make-believe play in the classroom is limited to one area only and that children are discouraged from using materials from other centers as props in their play. A more holistic approach to evaluation that includes parents’ and teachers’ views might contribute to a more favorable opinion of play-based curricula among school administrators and policymakers.

## 4. Not just child’s play

All SEL interventions seem to share some common concerns in regard to using play. One is an apparent contradiction between the child-controlled character of play and the adult role in supporting and enhancing play. With children coming to preschool and already playing at a mature level, the role of adults was limited until recently to providing the conditions (time, space, and materials) for play to happen; but it is no longer the case. Even when provided time, space, and materials, children playing with no adult support demonstrate a lower quality of play compared to children who receive some play tutoring from an adult delivered in the form of prompting, verbalization, and modeling (Kalkusch et al., 2021). Sometimes children even regress in their quality of play (Farran and Son-Yarbrough, 2001). At the same time, too much adult intervention in play destroys its voluntary and intrinsically motivated flow and thus potentially diminishes its potential benefits (Gmitrová and Gmitrov, 2003; Nome, 2015).

Also, play-based interventions cannot be completely formalized and manualized (Murphy and Gutman, 2012). While adults can and must create conditions for play, they cannot completely predict or control the result that emerges. Therefore, implementation fidelity cannot be reduced to teachers faithfully following the steps of the activities specified in the manual.

The analysis of play-based SEL interventions also highlights some of the challenges facing most of the interventions designed to be implemented in a classroom or any educational context: on the one hand, teachers should be given the freedom to adjust, modify, and individualize, but on the other hand, too much freedom makes it difficult to compare different classrooms and generalize the effect of the intervention. In addition, assigning teachers to a treatment group at random, as it is common in the evaluations of SEL interventions, may result in a poor fit between these teachers’ prior experiences and educational philosophy on the one hand and the nature of the intervention on the other. This might lead to these teachers’ low ‘commitment to implement’ (Cramer et al., 2021) and, in turn, to disappointing outcomes of the intervention. Although many of the education clearinghouses still use the results of the RCTs to select promising interventions, more and more educational researchers are now voicing their concerns about the preferential treatment of one particular research design and even about the appropriateness of the use of this ‘gold standard’ in education (Sullivan, 2011; Thomas, 2016).

In this paper, we examined some relationships between children’s play and SEL interventions. Given the multifaceted nature of play and the multitude of interventions designed to promote children’s social and emotional development, there could be many more relationships waiting to be examined. We propose that researchers pay attention to the quantity and quality of play while planning, implementing, or evaluating SEL interventions targeting young children. We expect that this might not only increase the effectiveness of these interventions but also contribute to our growing understanding of children’s development and learning.

## Data availability statement

The original contributions presented in the study are included in the article/Supplementary material, further inquiries can be directed to the corresponding author.

## Author contributions

All authors listed have made a substantial, direct and intellectual contribution to the work, and approved it for publication.

## Conflict of interest

The authors declare that the research was conducted in the absence of any commercial or financial relationships that could be construed as a potential conflict of interest.

## Publisher’s note

All claims expressed in this article are solely those of the authors and do not necessarily represent those of their affiliated organizations, or those of the publisher, the editors and the reviewers. Any product that may be evaluated in this article, or claim that may be made by its manufacturer, is not guaranteed or endorsed by the publisher.

## Footnotes

^1^In this paper, we are going to use ECE as one of the two most commonly used terms describing this field. In some of the literature reviewed the term ECEC is used, which stands for Early Childhood Education and Care.

^2^The term *spontaneous* is used in this context to mean *child-initiated* or *child-controlled*, which does not imply that play *spontaneously emerges* once a child reaches a certain age. In fact, if and when a specific kind of play emerges in children varies significantly among cultures and historical periods (Elkonin, 2005; Gaskins, 2014; Wood, 2014).
